# Direct and Indirect Costs of Cancer in Adult Population of Poland in the Period 2021–2023

**DOI:** 10.3390/cancers17233725

**Published:** 2025-11-21

**Authors:** Izabela Gąska, Aleksandra Czerw, Monika Pajewska, Olga Partyka, Dorota Charkiewicz, Andrzej Deptała, Anna Badowska-Kozakiewicz, Katarzyna Sygit, Ireneusz Dziubek, Paulina Wojtyła-Buciora, Jarosław Drobnik, Piotr Pobrotyn, Dorota Waśko-Czopnik, Tomasz Sowiński, Julia Pobrotyn, Adam Wiatkowski, Ewa Bandurska, Weronika Ciećko, Elżbieta Grochans, Anna Maria Cybulska, Daria Schneider-Matyka, Kamila Rachubińska, Tomasz Czapla, Karolina Kamecka, Remigiusz Kozlowski

**Affiliations:** 1 Medical Institute, Jan Grodek State University in Sanok, 38-500 Sanok, Poland; 2 Department of Health Economics and Insurance, Center for the Humanities and Social Sciences of Medicine, Medical University of Warsaw, 00-581 Warsaw, Poland; 3 Department of Economic and System Analyses, National Institute of Public Health NIH-National Research Institute, 00-791 Warsaw, Poland; 4 Department of Oncology Propaedeutics, Medical University of Warsaw, 01-445 Warsaw, Poland; 5 Faculty of Medicine and Health Sciences, University of Kalisz, 62-800 Kalisz, Poland; 6 Department of Family Medicine, Faculty of Medicine, Wroclaw Medical University, 51-141 Wroclaw, Poland; 7 Pulsantis Specialist and Rehabilitation Clinic Ltd., 53-238 Wroclaw, Poland; 8 Department of Gastroenterology, Hepatology with Inflammatory Bowel Disease Subunit, Provincial Specialist Hospital J. Gromkowskiego, 51-149 Wroclaw, Poland; 9 Department of Non-Surgical Clinical Sciences, Faculty of Medicine, Wroclaw University of Science and Technology, 50-370 Wroclaw, Poland; 10 Endocare Medical Center, Simple Joint-Stock Company (S.J.S.C.), 50-558 Wroclaw, Poland; 11 Faculty of Medicine, Wroclaw Medical University, 50-345 Wroclaw, Poland; 12 Center for Competence Development, Integrated Care and e-Health, Medical University of Gdansk, 80-204 Gdansk, Poland; 13 Department of Nursing, Faculty of Health Sciences, Pomeranian Medical University in Szczecin, 71-210 Szczecin, Poland; 14 Department of Management, Faculty of Management, University of Lodz, 90-237 Lodz, Poland; 15 Department of Management and Logistics in Healthcare, Medical University of Lodz, 90-131 Lodz, Poland

**Keywords:** cancer, costs, indirect costs, adults

## Abstract

Background/Objectives: Cancer is the second most common cause of death in Poland, responsible for 26.7% of deaths in 2023. Our aim was to estimate total direct and indirect costs of cancer in Poland. Methods: We acquired epidemiological data from the System Analysis and Implementation Database provided Polish Ministry of Health. For estimating the direct costs of medical procedures and chemotherapy, we used statistics of homogeneous groups of patients provided by the Polish National Health Fund. For analyzing the indirect costs in the field of absenteeism we acquired data from Statistical Portal of Polish Social Insurance Institution. Results: The total direct costs of treatment for cancer in the adult population in the period 2021–2023 to be equal to USD 192,935,858, taking purchasing power parity of Polish currency into account. This was equal to 0.00394% of General Domestic Product. The costs associated with absenteeism were equal to USD 3,103,526,321, which was equal to 0.063% of GDP. The sum of Years of Potential Life Lost was equal to 930,962 and the sum of Years of Potential Productive Life Lost was equal to 363,511. Cancer morbidity and incidence in the period 2021–2023 increased by 1.3%. Mortality increased by 2.1%. The total cost of medical services relative to GDP in 2022 was lower than in 2021; in 2023, however, it was higher than both in 2021 and 2022. The cost of chemotherapy, both in absolute numbers and relative to GDP, was significantly lower in 2022 than in 2021. Conclusions: In 2023, the cost was higher than in 2021 and in 2022. The costs of absenteeism in three consecutive years increased. Years of Potential Life Lost and Years of Potential Productive Life Lost due to cancer increased substantially in the period 2021–2023, both for males and for females, in all three age groups.

## Simple Summary

Cancer remains a major public health issue in Poland, ranking as the second leading cause of death and accounting for 26.7% of all deaths in 2023. This study evaluated both direct and indirect costs of cancer in the adult population between 2021 and 2023, using data from the Polish National Health Fund, the Ministry of Health, and the Social Insurance Institution. Direct treatment costs reached USD 192.9 million (PPP), equal to 0.0039% of GDP, while absenteeism related indirect costs were much higher at USD 3.1 billion (0.063% of GDP). Cancer cases increased by 1.3% during this period, and mortality rose by 2.1%. Years of Potential Life Lost totaled 930,962, and Years of Potential Productive Life Lost reached 363,511, both of which showed notable increases. The burden was evident in rising absenteeism and treatment expenses, particularly chemotherapy. These results highlight the growing health and economic impact of cancer in Poland and the urgent need for effective prevention and management strategies.

## 1. Introduction

Cancer is the second most common cause of death in Poland. According to the data provided by the Polish Ministry of Health [1], cancer was responsible for 19.61% of deaths in 2021, 23.62% of deaths in 2022, and 26.7% of deaths in 2023. According to information provided by the World Health Organization [2] 5-year prevalence of cancer in Poland was equal to 621,252 cases. The age-standardized incidence rate for 2022 was 262.8/100,000, and the age-standardized mortality rate for 2022 was 133.1/100,000. The cancers that were both the most common and leading reasons for death were lung, colorectal, and breast cancers. The cumulative risk of developing cancer before the age of 75 years was equal to 26.9% and the cumulative risk of dying from cancer before the age of 75 years was equal to 14.5%. What is more, the estimations provided by the International Agency for Research on Cancer show expected growth regarding the number of new cases [3]. For the year 2045, 26.9% more cases are expected when compared to 2022. As for mortality, 35.8% more cases are expected when compared to 2022.

The main risk factors associated with lifestyle and environment are obesity, infections, UV radiation, and alcohol [4,5,6]. According to Global Cancer Observatory [7], 6.7% cancer cases in Poland are attributable to excessive body mass index, age-standardized rate for cancers attributable to infections was equal to 18.1/100,000, population attributable fraction of all cancer cases in Europe attributable to ultraviolet radiation exposure was equal to 1%, age-standardized rate for cancers attributable to alcohol drinking was equal to 12.2/100,000. At the same time, 0.34% of cancer cases could have been prevented if the mean body mass index had remained constant as it was back in 1982.

Diagnostic and screening tests for cancer are either image-based, e.g., mammography, colonoscopy, computed tomography, or biospecimen-based, e.g., HPV testing, fecal occult blood testing, prostate-specific antigen [8]. The treatment methods of cancer vary by cancer type and stage; mostly, it is based on a combination of treatments, surgery, chemotherapy, and radiation therapy [9]. Other treatment types include hormone therapy, immunotherapy, blood stem cell transplant, and targeted therapy.

Cancer, a chronic disease, requires long-term treatment and usually limits the social and professional life of patients. What is more, the treatment is usually expensive. Entire families are affected by the diagnosis of cancer, which causes emotional distress but also requires the family members to be involved in caregiving responsibilities. The patients tend to withdraw from their social activities because of the physical weakness, emotional distress, and side effects of the treatment. Therefore, their social lives are affected, and their quality of life diminishes. The diagnosis of cancer impacts the financial situation of the patients, and their families. The cancer survivors experience worsened financial status both during and after treatment, even if assistance is provided. This negative phenomenon in the literature is referred to as financial toxicity [10,11,12].

Because of the costs of the treatment, the role of the public medical services is crucial. There are two main sources of financing public healthcare in Poland [13]: the National Health Fund and the national budget resources. The National Health Fund is based on health insurance premiums collected by two institutions, i.e., the Social Insurance Institution and the Agricultural Social Insurance Fund. Medical services and health insurance for people who do not have any income are covered by the state. The National Health Fund collects the budget and contracts healthcare services with institutions and providers. Private financing sources, like household resources, private health insurance, or charities, are also important as they cover close to one-third of healthcare expenditures [14]. As for the public resources health expenditures in 2023 in Poland were equal to 10.5% of total government expenditures, which is lower than the average for OECD countries, equal to 15.5% [15]. At the same time, the cancer mortality rate was equal to 240.4 and was higher than the average for OECD countries, equal to 201.6. Also, the risk factors like alcohol consumption were higher (11.0 liters per capita vs. 8.6 average for OECD), spending at least 150 min per week on physical activity was lower (20.3% vs. 40.3% average for OECD), and the change in air pollution mortality was lower as well (−12.3% vs. −31.3% average for OECD).

As for the economic burden of a disease, including cancer, two types of costs, i.e., direct and indirect costs, should be considered. The direct costs include the costs of treatment, both the public healthcare system’s expenditures and the patients’ out-of-pocket costs. The indirect costs include the costs of lost productivity due to sickness absence or presenteeism and years of life lost due to premature death [16]. Since, according to the Global Cancer Observatory, the incidence of cancer is expected to grow, a higher number of patients, higher treatment costs, and growing loss productivity due to sickness-absences and presenteeism are expected as well.

This article aims to estimate both the direct and indirect costs of cancer in Poland, in general, and not on specific types of cancer.

General Domestic Product in Poland in 2025 was equal to USD 1.08 trillion in 2025, which places it in the top 20 economies globally. Gross domestic product converted by purchasing power parity (PPP) conversion factor is growing as well from USD 46.1 K in 2022 through 46.8 K in 2023 to USD 50.4 K in 2024 [17]. However, at the same time poverty headcount ratio at national poverty line according to World Bank data has been growing in recent years, from 11.7% in 2022, through 12.2% in 2023 to 13.3% in 2024, and the gross domestic savings are falling from 23.7% of GDP in 2022, through 23.4% to 21.7% of GDP in 2023. The ratio of assets of pension funds providing retirement income to GDP was equal to 6.43% in 2020.

## 2. Materials and Methods

We acquired epidemiological data from the System Analysis and Implementation Database provided Polish Ministry of Health [1]. For estimating the direct costs of medical procedures and chemotherapy, we used statistics of homogeneous groups of patients provided by the Polish National Health Fund [18]. Polish National Health Fund contracts all medical procedures and the providers in order to be paid report the precise costs of medical procedures. Therefore, the data in the system are precise amounts and not estimates. However, because not all types of costs are available, we call the results provided in our paper estimates, to underline limitations, which are consequences of the types of costs that were not evaluated and included in the analysis. The statistics of homogeneous groups of patients are based on categories of inpatient medical services and not on ICD diagnostic codes. Therefore, for clarity and precision, we list the medical services included in our analysis. We based our estimates on the set of 21 categories of medical services, i.e., Comprehensive Intracranial Procedures For Malignant Cancer Diagnosis, Brain And Spinal Cord Tumors, Extensive Oral, Pharyngeal, And Laryngeal Cancer Surgery With Reconstruction, Comprehensive Oral, Pharyngeal, And Laryngeal Cancer Surgery For Malignant Cancer Diagnosis, Respiratory And Thoracic Cancer Diseases, Major And Endoscopic Colon Cancer Surgery, Major Abdominal Surgery For Malignant Cancer Diagnosis, Biliary Tract Cancer, Resective Surgery For Malignant Or Tumorous Lesions With Arthritis Plasma, Mid-Sized Soft Tissues For Malignant Cancer Diagnosis, Minor Musculoskeletal Or Soft Tissues For Malignant Cancer Diagnosis, Major Surgery For Breast Area In The Diagnosis Of Malignant Cancer, Malignant Breast Diseases, Radical Surgery For Endocrine Cancer, Extra-Pituitary Endocrine Cancer, Endocrine Cancer Qualification For Radioactive Iodine Treatment Of Thyroid Cancer, Kidney And Urinary Tract Cancer, Radical Prostatectomy, Conservative Treatment Of Malignant Reproductive Cancer, Intensive Treatment Of Acute Leukemias and Comprehensive Oncological Diagnosis. Chemotherapy is calculated as a separate category, as it is provided as a separate category in the system for statistics of homogeneous groups of patients as well. Polish National Health Fund provides the amount of public money spent for each category in PLN. Therefore, our analysis is focused on public healthcare costs only. We converted each amount to international $ using purchasing power parity (PPP) conversion factors provided by the World Bank Group [19]. For three consecutive years, 2021, 2022, and 2023, they were equal to 1.75, 1.83, and 1.99, respectively. We divided the amount in PLN by these conversion factors. Also, we converted the costs into fractions of General Domestic Product values. We used the data provided by the national Central Statistical Office [20] for three consecutive years; however, for 2023, only a preliminary estimate is available. We divided the costs in each category by General Domestic Product values, both in PLN and expressed the ratio as percentages.

**Figure 1:**
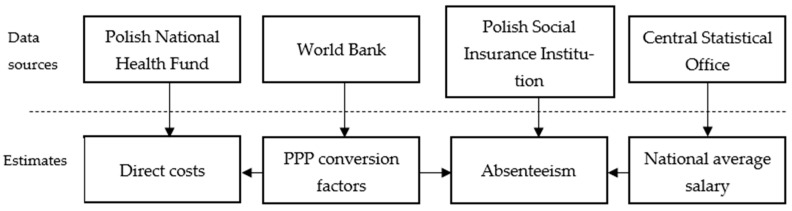
Summary of data sources and the calculated estimates.

For analyzing the indirect costs in the field of absenteeism we acquired data from Statistical Portal of Polish Social Insurance Institution [21]. The loss due to absenteeism was estimated by multiplying the number of days on a sick leave from the from actual social insurance claims by the national average salary, which according to Central Statistical Office, but after conversion on the basis of purchasing power parity, was equal to $PPP 3247.41, $PPP 3467.84, and $PPP 3595.72 for 2021, 2022, and 2023, respectively. The aforementioned calculations are summarized in Figure 1.

Finally, we calculated the sums of Years of Potential Life Lost (YPLL) and Potential Productive Life Lost (YPPLL). We based our calculations on the average life expectancy and the number of deaths attributable to cancer, both provided by the Central Statistical Office for 2021, 2022, and 2023 [20]. To calculate Years of Potential Life Lost, we subtracted the number of years lived from the average life expectancy for each age group and multiplied the difference by the number of deaths attributable to cancer for each age group. Similarly, to calculate Years of Potential Productive Life Lost, we calculated the number of years lived from the point of entering the workforce and subtracted it from the average life expectancy till the age of retirement. We did this for each age group, separately for females and for males, and multiplied the difference by the number of deaths attributable to cancer for each age group. The age of retirement in Poland is 60 years old for females and 65 years old for males; therefore, separate calculations for males and for females were necessary.

For all data included, we decided to focus on the period 2021–2023 because the Polish National Health Fund changed the system of medical service categories, so they are partially different for 2021 and beyond from the system that was in use before 2021. Also, we limited our calculations to data provided for adults.

## 3. Results

From the epidemiology aspect of cancer, Table 1 depicts morbidity, incidence, and mortality due to cancer in Poland for the period 2021–2023, both in absolute numbers and per 100,000 population members.

As for morbidity, the number of cancer cases increased from 1,970,519 in 2021 to 1,996,412 in 2023, which means it increased by 1.3%. Incidence increased from 1,188,080 in 2021 to 1,203,256 in 2023, which also means it increased by 1.3%. Mortality, however, increased from 123,381 in 2021 to 125,939 in 2023, which means it increased by 2.1%. The mortality increased more than morbidity and incidence in the period of three consecutive years.

Regarding the public economic burden, Table 2 depicts the value of 22 categories of medical services reimbursed by the National Health Fund with the use of purchasing power parity (PPP) US dollars as the unit of currency. Also, the percentage values relative to Gross Domestic Product for three consecutive years are provided.

Total cost of medical services in absolute values was similar in 2021 and in 2022, then in 2023 it was significantly higher. The total cost of medical services relative to GDP in 2022 was lower than in 2021; in 2023, however, it was higher than in both 2021 and 2022. The cost of chemotherapy both in absolute numbers and relative to GDP was significantly lower in 2022 than in 2021. In 2023, the cost was higher than in 2021 and in 2022. The cost relative to GDP in 2023 was higher than in 2022, but lower than in 2021.

Table 3 depicts the costs of absenteeism attributable to sick leaves due to cancer, both in $PPP and as a percentage of General Domestic Product.

The costs of absenteeism in three consecutive years increased. As a percentage of Gross Domestic Product, the costs were higher in 2023 than in 2021 and 2022.

Table 4 depicts the Years of Potential Life Lost and Years of Potential Productive Life Lost due to cancer for males and for females for the period 2021–2023.

Years of Potential Life Lost and Years of Potential Productive Life Lost due to cancer increased substantially in the period 2021–2023, both for males and for females, in all three age groups. The total increase regarding Years of Potential Life Lost in the period 2021–2023 was equal to 9.1%. The total increase regarding Years of Potential Productive Life Lost in the period 2021–2023 was equal to 17.8%.

## 4. Discussion

The majority of papers published focus on the costs of specific cancer [22,23,24,25,26]. We attempted to quantify the costs associated with cancer and general. However, similar attempts have been made as well. The mean total cost of cancer in Malaysia was estimated to be equal to USD 1893.46 per patient per year, with direct non-medical costs being the largest contributor, accounting for 46.1% of the total cost [27]. Indirect costs accounted for over 50% of the total economic burden of most cancers in Taiwan [28]. The top 10 high-expenditure cancers regarding both direct medical costs, and indirect costs were lung cancer, female breast cancer, colorectal cancer, liver cancer, oral cancer, leukaemia, prostate cancer, non-Hodgkin’s lymphoma, gastric cancer, and oesophageal cancer. The financial costs of informal cancer care in Canada were estimated to be equal to USD 4809 per month on average [29]. It was found to be highest in the final, palliative stage of the disease. However, a review on the topic found palliative care to be cost effective [30]. In Iran, the average cost of cancer treatment was estimated to be equal to USD 73,278 per patient. The insurance companies covered 87%, the governmental share was 6% and 7% was out of pocket cost [31].

A study with methodology close to the one we used in the current paper [32], i.e., estimating costs of cancer on the basis of official data sources from public health and social care providers, found the economic impact of cancer in Chile to be higher than 2100 million dollars a year, which is close to 1% of the Gross Domestic Product of the country. The indirect costs were estimated to be 1.92 times higher than the direct costs.

According to our estimations, the total direct costs of treatment for cancer in the adult population in the period 2021–2023 were equal to $PPP 192,935,858, which was equal to 0.00394% of General Domestic Product. The costs associated with absenteeism were equal to $PPP 3,103,526,321, which was equal to 0.063% of GDP. The sum of Years of Potential Life Lost was equal to 930,962 and the sum of Years of Potential Productive Life Lost was equal to 363,511.

However, our results have several limitations. First, public financing regarding outpatient specialist care in Poland is complex and varies because various units have specific individual contracts. These costs were not included in the current analysis. Secondly, disability weights for cancer depend on the cancer type, and stage and there is no single mean value for cancer in general, which impedes calculating the costs of presenteeism and Disability-Adjusted Life Years. Also, the official resources do not provide the costs of medical devices and transportation, additional medications, physiotherapy, nursing home costs, or palliative care. No estimations for out-of-pocket costs regarding treatment for cancer, in general, or the costs of family caregivers are available in Poland. Still, these costs are not negligible and can lead to financial catastrophe for patients [33]. The meta-analysis [34] showed the pooled prevalence of catastrophic health expenditure to be equal to 47% in middle- and high-income countries, and to 74.4% in low-income countries. To sum up, we must recognize that our estimates do not include all costs associated with cancer.

## 5. Conclusions

From cancer morbidity and incidence in the period 2021–2023, the number of cancer cases in Poland increased by 1.3%. Mortality, however, increased by 2.1%, which means that the mortality increased more than morbidity and incidence in the period of three consecutive years.

The total cost of medical services in absolute values was similar in 2021 and in 2022, then in 2023 it was significantly higher. The total cost of medical services relative to GDP in 2022 was lower than in 2021; in 2023, however, it was higher than in both in 2021 and 2022. The cost of chemotherapy both in absolute numbers and relative to GDP, was significantly lower in 2022 than in 2021. In 2023, the cost was higher than in 2021 and in 2022. The drop in costs in 2022 may be a consequence of limited access to medical services and diagnosis during the COVID-19 pandemic [35]. This temporary drop may lead to much higher costs in the future, because of the negligence in screening and providing treatment during the pandemic from both the medical service system and the patients who could delay seeking healthcare.

The costs of absenteeism in three consecutive years increased. Years of Potential Life Lost and Years of Potential Productive Life Lost due to cancer increased substantially in the period 2021–2023, both for males and for females, in all three age groups.

General Domestic Product in Poland in consecutive years is growing, but at the same time poverty headcount ratio is growing as well, while gross domestic savings are falling. This may suggest increased diversity regarding patients’ economic situation. At the same time, cancer morbidity and incidence is rising as well as the associated costs. This calls for applying effective policy, including effective screening for cancer, prevention targeted at risk factors, and securing adequate financial resources for the treatment.

## Figures and Tables

**Table 1:** Morbidity, incidence, and mortality due to cancer in Poland for the period 2021–2023.

	Unit	2021	2022	2023	2021–2023	
Morbidity	absolute numbers	1,970,519	1,986,335	1,996,412	Total	5,953,266
	per 100,000	5136.75	5181.53	5210.14	Mean	5176.14
Incidence	absolute numbers	1,188,080	1,197,334	1,203,256	Total	3,588,670
	per 100,000	3097.51	3123.96	3140.93	Mean	3120.80
Mortality	absolute numbers	123,381	124,660	125,939	Total	373,980
	per 100,000	323.90	327.27	330.63	Mean	327.27

Source: Calculations based on the data from System Analysis and Implementation Database. Ministry of Health.

**Table 2:** Public direct costs associated with treatment of cancer.

	2021		2022		2023		Total	
Direct Costs	PPP$	%GDP	PPP$	%GDP	PPP$	%GDP	PPP$	%GDP
Comprehensive Intracranial Procedures For Malignant Cancer Diagnosis	344,829	0.00002	1,822,456	0.00011	2,511,336	0.00015	4,804,356	0.00010
Brain And Spinal Cord Tumors	568,131	0.00004	748,251	0.00004	2,028,976	0.00012	3,441,500	0.00007
Extensive Oral, Pharyngeal, And Laryngeal Cancer Surgery With Reconstruction	2,226,707	0.00015	3,007,118	0.00018	3,832,209	0.00022	9,153,688	0.00019
Comprehensive Oral, Pharyngeal, And Laryngeal Cancer Surgery For Malignant Cancer Diagnosis	98,946	0.00001	144,252	0.00001	139,261	0.00001	384,014	0.00001
Respiratory And Thoracic Cancer Diseases	4,864,102	0.00032	1,984,185	0.00012	772,769	0.00005	7,355,402	0.00015
Major And Endoscopic Colon Cancer Surgery	1,420,987	0.00009	1,599,895	0.00010	1,949,736	0.00011	4,997,048	0.00010
Major Abdominal Surgery For Malignant Cancer Diagnosis	920,802	0.00006	1,013,992	0.00006	1,374,160	0.00008	3,334,187	0.00007
Biliary Tract Cancer	480,634	0.00003	599,357	0.00004	905,584	0.00005	2,010,777	0.00004
Resective Surgery For Malignant Or Tumorous Lesions With Arthritis Plasma	14,841,035	0.00099	18,801,462	0.00112	22,021,887	0.00129	56,022,603	0.00114
Mid-Sized Soft Tissues For Malignant Cancer Diagnosis	50,537	0.00001	83,930	0.00001	121,645	0.00001	260,272	0.00001
Minor Musculoskeletal Or Soft Tissues For Malignant Cancer Diagnosis	2401	0.00001	9222	0.00001	4317	0.00001	15,951	0.00001
Major Surgery For Breast Area In The Diagnosis Of Malignant Cancer	105,002	0.00001	368,862	0.00002	509,501	0.00003	1,006,817	0.00002
Malignant Breast Diseases	2,035,688	0.00014	3,181,295	0.00019	4,649,514	0.00027	10,019,762	0.00020
Radical Surgery For Endocrine Cancer	5,587,039	0.00037	224,927	0.00001	379,477	0.00002	5,883,921	0.00012
Extra-Pituitary Endocrine Cancer	2,280,745	0.00015	1,586,989	0.00009	1,751,063	0.00010	5,580,703	0.00011
Endocrine Gland Tumors	478,334	0.00003	1,227,374	0.00007	990,056	0.00006	2,716,876	0.00006
Endocrine Cancer Qualification For Radioactive Iodine Treatment Of Thyroid Cancer	2,669,737	0.00018	3,834,971	0.00023	4,699,688	0.00027	11,313,127	0.00023
Kidney And Urinary Tract Cancer	6,732,015	0.00045	7,152,315	0.00043	4,252,806	0.00025	17,920,885	0.00037
Radical Prostatectomy	599,152	0.00004	593,056	0.00004	260,443	0.00002	1,425,855	0.00003
Conservative Treatment Of Malignant Reproductive Cancer	3,794,477	0.00025	1,312,231	0.00008	5,830,319	0.00034	11,098,953	0.00023
Intensive Treatment Of Acute Leukemias	9,470,407	0.00063	11,148,779	0.00066	13,240,496	0.00077	34,045,197	0.00070
Comprehensive Oncological Diagnosis	17,901	0.00001	25,240	0.00001	95,607	0.00001	143,963	0.00001
**Total**	**59,589,607**	**0.00396**	**60,470,158**	**0.00360**	**72,320,851**	**0.00423**	**192,935,858**	**0.00394**
**Chemotherapy**	**283,531,150**	**0.01886**	**275,628,160**	**0.01640**	**309,147,761**	**0.01808**	**868,700,586**	**0.01774**

Source: Calculations based on the data from National Health Fund data.

**Table 3:** Indirect costs regarding absenteeism associated with cancer.

Year		Days/Numbers	$PPP	%GDP
2021	Sick leave days	8,574,932	928,211,072.53	0.062%
	Sick leave numbers	428,691		
2022	Sick leave days	9,061,963	1,047,515,054.51	0.062%
	Sick leave numbers	479,553		
2023	Sick leave days	9,443,696	1,131,895,776.45	0.066%
	Sick leave numbers	523,953		
Total	Sick leave days	27,080,591	3,103,526,320.78	0.063%
	Sick leave numbers	1,432,197		

Source: Calculations based on the data from Polish Social Insurance Institution, Central Statistical Office.

**Table 4:** Years of Potential Life Lost and Years of Potential Productive Life Lost due to cancer for males and for females for the period 2021–2023.

		YPLL			YPPLL		
	Age	2021	2022	2023	2021	2022	2023
Males	30	5946	6536	6027	1081	1391	1407
	45	26,141	31,170	30,181	8181	10,890	11,241
	60	109,590	115,866	121,125	77,880	84,836	90,210
	Total	141,677	153,572	157,333	87,142	97,117	102,858
Females	30	4729	5121	4631	1909	2151	1991
	45	36,726	39,751	41,670	21,351	23,731	25,260
	60	111,318	116,653	117,781	-	-	-
	Total	152,773	161,525	164,083	23,260	25,882	27,252
Total	30	10,676	11,657	10,659	2991	3542	3399
	45	62,867	70,921	71,851	29,532	34,621	36,501
	60	220,908	232,519	238,906	77,880	84,836	90,210
	Total	294,450	315,097	321,415	110,402	122,999	130,110

Source: Calculations based on the data from Central Statistical Office.

## Data Availability

Data available from authors.
